# Prognostic value of myocardial strain and late gadolinium enhancement on cardiovascular magnetic resonance imaging in patients with idiopathic dilated cardiomyopathy with moderate to severely reduced ejection fraction

**DOI:** 10.1186/s12968-018-0466-7

**Published:** 2018-06-14

**Authors:** Seung-Hoon Pi, Sung Mok Kim, Jin-Oh Choi, Eun Kyoung Kim, Sung-A Chang, Yeon Hyeon Choe, Sang-Chol Lee, Eun-Seok Jeon

**Affiliations:** 1 Department of Internal Medicine, Heart Vascular Stroke Institute, Samsung Medical Center, Sungkyunkwan University School of Medicine, 81 Irwon-ro, Gangnam-gu, Seoul, 06351 Republic of Korea; 2 Department of Radiology, Cardiovascular Imaging Center, Samsung Medical Center, Sungkyunkwan University School of Medicine, Seoul, Republic of Korea

**Keywords:** Cardiovascular magnetic resonance imaging, Myocardial strain, Prognosis, Late gadolinium enhancement, Idiopathic dilated cardiomyopathy

## Abstract

**Background:**

It has been reported that left ventricular (LV) myocardial strain and late gadolinium enhancement (LGE) on cardiovascular magnetic resonance (CMR) imaging have prognostic value in patients with heart failure (HF). However, previous studies included patients with various systolic functions. This study aimed to investigate the prognostic value of LV myocardial strain and LGE on CMR imaging in patients with idiopathic dilated cardiomyopathy (DCM) with reduced ejection fraction (EF < 40%).

**Methods:**

From a prospectively followed cohort who underwent CMR between November 2008 and December 2015, subjects with LV EF < 40% and a diagnosis of idiopathic DCM were eligible for this study. The CMR images were analyzed for LV and right ventricular (RV) function, presence and extent of LGE, and LV myocardial strain. The primary outcome was a composite of all-cause death and heart transplantation. The secondary outcome was hospitalization for HF.

**Results:**

A total of 172 patients were included, in whom mean LV EF was 23.7 ± 7.9% (EF 30–40% *n* = 47; EF < 30% *n* = 125). During a median follow-up of 47 months, the primary outcome occurred in 43 patients (16 heart transplantations, 29 all-cause deaths), and there were 41 hospitalizations for HF. Univariate Cox proportional hazard regression analysis showed that mean arterial pressure, serum sodium concentration, log of plasma NT-proBNP level, and presence of LGE (HR 2.277, 95% CI: 1.221–4.246) were significantly associated with the primary outcome. However, LV strain had no significant association (HR 1.048, 95% CI: 0.945–1.163). Multivariable analysis showed that presence of LGE (HR 4.73, 95% CI: 1.11–20.12) and serum sodium (HR 0.823, 95% CI: 0.762–0.887) were independently associated with the primary outcome.

**Conclusions:**

LGE in CMR imaging was a good predictor of adverse outcomes for patients with idiopathic DCM and reduced EF. Identification of LGE could thus improve risk stratification in high-risk patients. LV strain had no significant prognostic value in patients with moderate to severe systolic dysfunction.

**Electronic supplementary material:**

The online version of this article (10.1186/s12968-018-0466-7) contains supplementary material, which is available to authorized users.

## Background

Idiopathic dilated cardiomyopathy (DCM) accounts for a substantial proportion of heart failure (HF) cases [1, 2]. It is associated with significant morbidity and mortality due to HF and sudden cardiac death [3–5]. Although several factors in patients with HF [6–15] are associated with an adverse prognosis, risk stratification remains challenging. Therefore, better tools are needed for risk stratification in order to guide individualized treatment strategies and patient surveillance.

Cardiovascular magnetic resonance (CMR) imaging has become recognized as the gold standard for assessment of cardiac function and mass [16, 17], and it can be used to distinguish the etiology of HF [18, 19]. Additionally, it has been reported that the appearance of late gadolinium enhancement (LGE) [20–23] and left ventricular (LV) myocardial strain in CMR imaging [24] have prognostic value in patients with non-ischemic DCM. However, those studies included patients with various systolic functions.

Therefore, we investigated the prognostic value of LV myocardial strain and LGE in the CMR images of patients with idiopathic DCM with reduced ejection fraction (EF).

## Methods

### Study population

From a prospectively followed cohort who underwent CMR at Samsung Medical Center, Seoul, Korea, between November 2008 and December 2015, subjects whose LV EF was less than 40% were eligible for this study (*n* = 441). Medical records were reviewed, and those from patients with an LV EF less than 40% who had been diagnosed previously with idiopathic DCM were evaluated. The diagnosis of DCM was made according to the criteria of the World Health Organization/International Society and Federation of Cardiology [25]. Patients had to exhibit dilatation and impaired contraction of the LV or both ventricles in the absence of valvular disease, hypertensive heart disease, and congenital abnormalities. The possibility of ischemic heart disease was excluded by invasive x-ray coronary angiography or non-invasive testing such as coronary computed tomography angiography (defined as ≥50% luminal stenosis) [24] or CMR itself (subendocardial or transmural pattern of LGE suggestive of previous myocardial infarction) [18], according to each physician’s clinical decision.

**Fig. 1:**
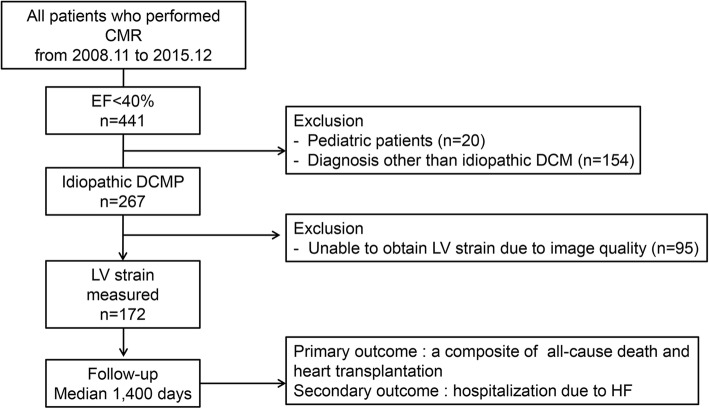
Study Design and Population. Abbreviations: CMR, cardiovascular magnetic resonance; DCM, dilated cardiomyopathy; EF, ejection fraction; HIV, human immunodeficiency virus; LV, left ventricular

Of all potentially eligible patients (*n* = 441), pediatric patients (*n* = 20) and 154 patients with ischemic heart disease, constrictive pericarditis, tachycardia-induced cardiomyopathy, cardiomyopathy due to endocrine dysfunction, cardiomyopathy due to infection with the human immunodeficiency virus, stress induced cardiomyopathy, infiltrative myocardial disease, hypertensive heart disease, cardiomyopathy due to systemic autoimmune disease, alcoholic cardiomyopathy, cardiomyopathy related to chemotherapeutic agents, restrictive cardiomyopathy, significant organic valvular disease, or hypertrophic cardiomyopathy were excluded. Of 267 patients with a diagnosis of idiopathic DCM, we excluded 95 because their CMR images were inappropriate for strain measurement, resulting in a final sample size of 172 patients (Fig. 1). This study and all of its analyses were approved by the Institutional Review Board of Samsung Medical Center which waived written informed consent.

### CMR image acquisition

All subjects underwent CMR in a 1.5 T scanner (Magnetom Avanto, Syngo MR B17 version; Siemens Healthineers, Erlangen, Germany) with a 32-channel phased-array receiver coil. CMR scans consisted of localizing images (axial, coronal, and sagittal), cine scans, and LGE scans. After localization, cine images of the LV were acquired using a balanced steady-state free-precession sequence in the 4-, 3-, and 2-chamber and short axis views to obtain contiguous slices that included the entire LV, with a 6-mm slice thickness and 4-mm intersection gaps. At each level, cine images were composed of 30 phases per cardiac cycle. Cine images were obtained during multiple breath-holds. LGE imaging was acquired using a phase-sensitive inversion recovery technique 10 min after injection of 0.2 mmol/kg gadobutrol (Gadovist; Bayer Healthcare, Berlin, Germany) at a rate of 3 ml/sec, followed by a 30-ml saline flush. Contiguous short-axis image acquisition of 10–12 slices was used, with 6 mm thickness and a 4-mm interslice gap. Inversion delay times were typically 280–360 msec.

### LV myocardial strain analysis

**Fig. 2:**
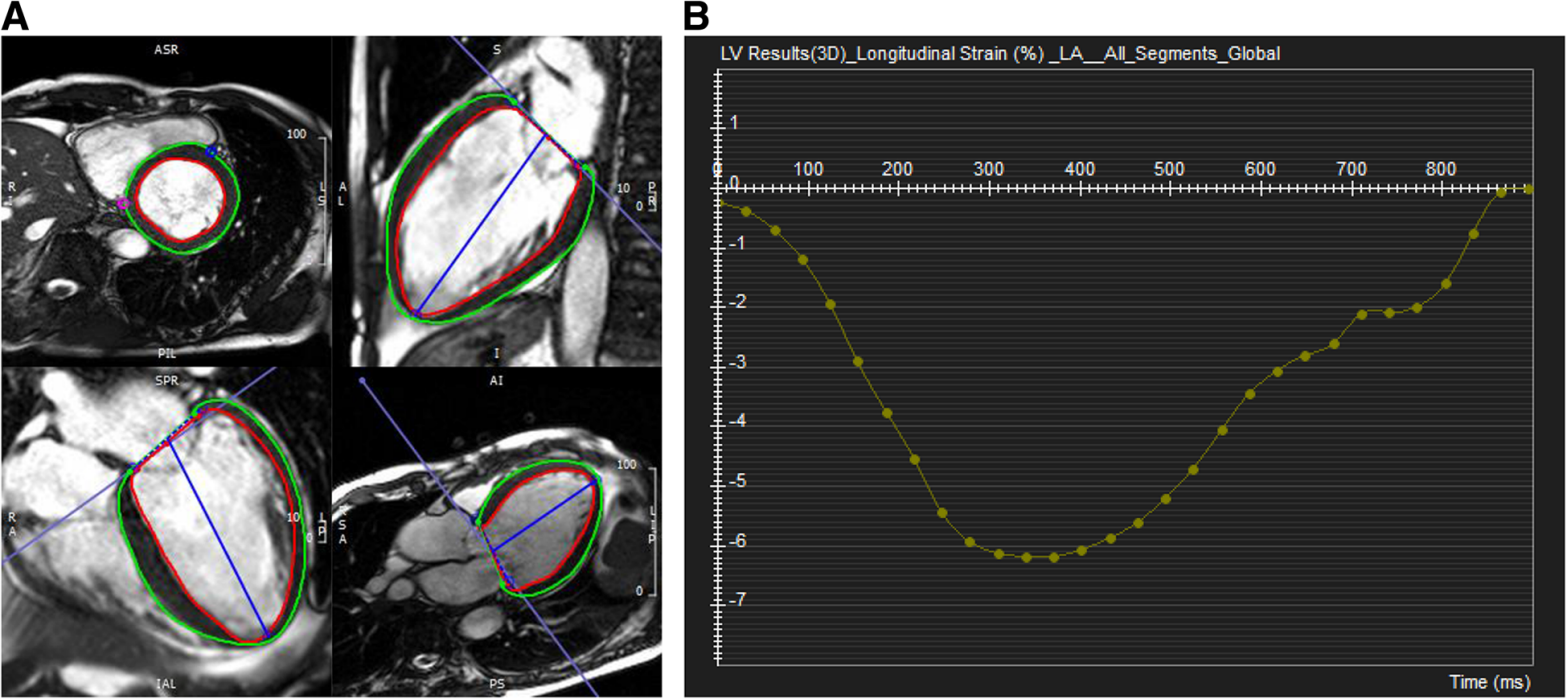
Strain Analysis by Feature Tracking. **a** The endocardial and epicardial borders were traced manually at end-diastolic phase. **b** Global peak longitudinal strain curve by feature tracking. Abbreviations: LA, left atrial; LV, left ventricular

CMR tissue tracking analyses were performed using commercially available software (cvi42 version 5, Circle Cardiovascular Imaging Inc., Calgary, Alberta, Canada). Two-, three-, and four-chamber and short axis images were uploaded into the software, which reconstructs a 3D model that we used to analyze 2D-radial, circumferential, and longitudinal LV strain. The preferred images were loaded into the analysis/viewer frame of the software and analyzed in random order by two investigators (SHP with 1 year and JWH with 3 years of CMR experience) who were independently blinded to the clinical findings. Tissue tracking analysis was manually performed by drawing the endo- and epicardial surfaces in end-diastolic phase (reference phase) using short axis stacked slices (Fig. 2). A short axis reference point was manually delineated at the right ventricle (RV) upper and lower septal insertion of the LV for regional and global analysis of strain and the generation of polar map views. Next, the software automatically drew the contour and traced its myocardium voxel points throughout the remainder of the cardiac cycle. The algorithm determined and depicted the left borders of the LV myocardium in the following phases of a cardiac cycle based on the endo- and epicardial contours of the reference phase. The software automatically performed 2D strain analyses of all slices.

The routine cine images of 172 patients included for strain analysis consist of 30 images per cardiac cycle. Those images were acquired using retrospective (electrocardiogram (ECG)-gated, multi-breathhold technique. The patients excluded from the analyses for strain underwent different scan technique using single-breathhold method for obtaining cine images because of arrhythmia or lack of breath-hold. Because of this, the number of image frames was variable (10~ 24 images), so the temporal resolution was also different and we could not perform the analysis of LV strain.

### LGE measurement

The presence and extent of LGE were evaluated by one observer experienced in LGE-CMR, who was blinded to clinical data and outcomes. For quantification of fibrosis, LGE was defined as areas with a signal intensity >6SD [26, 27] above the mean signal intensity of remote myocardium in the same short-axis slice using commercial software (CAAS MRV version 1.0, Pie Medical Imaging B.V., Maastricht, The Netherlands). Areas are expressed as mass and percentage of myocardial mass.

### Follow-up and endpoints

All patients were followed by medical record review. Vital status was cross-checked in all of the patients using National Insurance data from the Korean government, which contain unique identifiers for the patients [28]. The median follow-up duration was 1400 days (Q1–Q3: 770 to 2210 days). The primary outcome was a composite of all-cause death and heart transplantation. The secondary outcome was first hospitalization due to HF.

### Statistical analysis

Categorical variables are presented as numbers and relative frequencies (percentages), and continuous variables as means and standard deviations or medians with interquartile ranges (Q1–Q3), according to their distribution, which was checked using the Kolmogorov-Smirnov test. Categorical variables were compared using chi-square tests, and continuous variables were compared using Student’s t-test or analysis of variance. Cumulative events rates were calculated based on Kaplan-Meier censoring estimates, and the log-rank test was used to compare survival curves. We proceeded with multiple Cox regression model to understand the variability in time to event in the two primary endpoints. Based on univariate analysis, all demographic or clinical variables with *p*-values < 0.2 were initially considered to enter the model. Then, we eliminated insignificant variables (*p* > 0.05) one by one to obtain a robust and parsimonious model for prediction. We verified the model assumption of proportional hazards via Schoenfeld residuals. When the assumption fails, we attempted accommodating time-dependent covariates and stratified analyses. As a measure of goodness of the prediction model, we obtained the Harrel’s c-index. Pearson correlation coefficient was calculated to assess the correlation between LVEF and LV myocardial strains. Inter- and intra-observer variabilities for strain values were assessed by the repeated analysis of 30 randomly selected patients. Statistical analyses were performed using SAS version 9.3 (SAS Institute, Cary, North Carolina, USA), and SPSS (version 19.0, International Business Machines, Armonk, New York, USA). *P*-values < 0.05 (2-sided) were considered statistically significant.

## Results

### Baseline characteristics

The baseline clinical characteristics and CMR variables of all 172 patients are summarized in Tables 1 and 2. Also these data of those who were excluded from and included in the strain analysis was presented in the Additional file 1: Tables S1 and S2. There was no significant difference between 95 excluded and 172 included patients except gender, presence of LBBB, LV myocardial mass. There was no difference in the presence of LGE between the two groups. Mean LV EF of the study subjects was 23.7 ± 7.9% (EF 30–40% *n* = 47; EF < 30% *n* = 125) and LGE was observed in 66 (38.2%) patients. The Pearson correlation coefficients between LV EF and GLS, GCS, and GRS were − 0.733 (*P* < 0.01), − 0.780 (*P* < 0.01), and 0.739 (*P* < 0.01).

**Table 1:** Baseline characteristics and CMR parameters for patients with and without the primary outcome

*Parameters*	*All patients (n = 172)*	*Patients without the primary outcome (n = 129)*	*Patients with the primary outcome (n = 43)*	*P value*
Age (years)	56.4 ± 14.3	57.0 ± 14.6	54.8 ± 13.5	0.398
Male gender, n (%)	116 (67.4)	85 (65.9)	31 (72.1)	0.452
Mean arterial pressure (mmHg)	84 ± 13	86 ± 13	80 ± 12	0.015
Hypertension, n (%)	58 (33.7)	44 (34.1)	14 (32.6)	0.852
Diabetes mellitus, n (%)	36 (20.9)	25 (19.4)	11 (25.6)	0.387
Dyslipidemia, n (%)	11 (6.4)	8 (6.2)	3 (7.0)	0.857
Current smoker, n (%)	49 (28.5)	37 (28.7)	12 (27.9)	0.734
Chronic kidney disease^a^, n (%)	31 (18.0)	21 (16.3)	10 (23.3)	0.303
Previous CVA, n (%)	4 (2.3)	1 (0.8)	3 (7.0)	0.049
Body mass index (kg/m^2^)	24.0 ± 4.5	24.2 ± 4.5	23.5 ± 4.4	0.347
ECG at baseline				
Heart rate (bpm)	83 ± 20	84 ± 19	83 ± 22	0.883
Left bundle-branch block, n (%)	31 (18.0)	23 (17.8)	8 (19.0)	0.859
QRS duration (ms)	113 ± 29	112 ± 30	117 ± 29	0.305
Laboratory data				
Serum creatinine (mg/dl)	0.97 ± 0.27	0.96 ± 0.25	1.01 ± 0.31	0.247
Na (mmol/l)	139.6 ± 3.3	140.2 ± 2.8	137.6 ± 3.8	< 0.001
ln(NT-proBNP) (pg/ml)	7.22 ± 1.27	7.08 ± 1.29	7.60 ± 1.16	0.022
Cardiac medications				
Beta-blockers, n (%)	120 (69.8)	87 (67.4)	33 (76.7)	0.250
ACE-inhibitors/ARB, n(%)	134 (77.9)	104 (80.6)	39 (90.7)	0.126
Spironolactone, n (%)	101 (58.7)	74 (57.4)	27 (62.8)	0.531
Diuretics, n (%)	125 (72.7)	93 (72.1)	32 (74.4)	0.767
Digoxin, n (%)	33 (19.2)	21 (16.3)	12 (27.9)	0.094

Primary outcome: all-cause death, heart transplantation during follow-up. Values are mean ± SD, n(%)

^a^Chronic kidney disease was defined as eGFR < 60 ml/min/1.73m^2^, calculated using the 4-component MDRD study equation

Abbreviations: *ACE* angiotensin-converting-enzyme, *ARB* angiotensin II receptor blockers, *BNP* B-type natriuretic peptide, *CVA* cerebrovascular accident *ECG* electrocardiography

**Table 2:** Baseline standard CMR-data and myocardial deformation parameters of patients with and without the primary outcome

*Parameters*	*All patients (n = 172)*	*Patients without the primary outcome (n = 129)*	*Patients with the primary outcome (n = 43)*	*P value*
LV EF (%)	23.7 ± 7.9	23.6 ± 8.0	24.1 ± 7.5	0.675
LV EDV (ml)	284.3 ± 91.4	279.2 ± 81.5	299.9 ± 116.0	0.199
LV ESV (ml)	219.9 ± 85.7	215.5 ± 76.4	233.2 ± 109.0	0.242
Cardiac output (L/min)	5.04 ± 1.53	4.95 ± 1.51	5.32 ± 1.57	0.166
Cardiac index (L/min/m^2^)	2.95 ± 0.84	2.89 ± 0.82	3.12 ± 0.88	0.114
RV EF (%)	41.2 ± 17.0	42.3 ± 18.0	37.7 ± 12.8	0.129
RV EDV (ml)	145.6 ± 60.7	140.7 ± 56.3	160.3 ± 70.9	0.066
RV ESV (ml)	91.6 ± 56.7	87.1 ± 53.8	105.1 ± 63.3	0.071
RV cardiac output (L/min)	4.21 ± 1.33	4.14 ± 1.23	4.43 ± 1.59	0.227
Presence of LGE, n (%)	66 (38.4)	42 (33.1)	24 (58.5)	0.004
Myocardial mass (g)	142.2 ± 41.1	144.1 ± 43.6	136.5 ± 31.9	0.232
Quantitative LGE mass (g)	6.8 ± 14.5	5.6 ± 13.3	10.6 ± 17.2	0.093
LGE mass/LV myocardial mass (%)	4.7 ± 9.5	3.8 ± 8.6	7.4 ± 11.5	0.073
Global radial strain (%)	12.3 ± 5.6	12.5 ± 5.6	11.9 ± 5.8	0.563
Global circumferential strain (%)	−7.3 ± 3.1	−7.3 ± 3.1	−7.2 ± 3.2	0.869
Global longitudinal strain (%)	−7.1 ± 2.9	−7.2 ± 2.9	−6.9 ± 2.7	0.613

Primary outcome: all-cause death, heart transplantation. Values are mean ± SD, n(%)

Abbreviations: *CMR* cardiovascular magnetic resonance, *EDV* end-diastolic volume, *EF*, ejection fraction, *ESV* end-systolic volume, *LGE* late gadolinium enhancement, *LV* left ventricle, *RV* right ventricle

### Outcomes

During the follow-up period, the primary outcome occurred in 43 patients (16 heart transplantations, 29 all-cause deaths), and there were 41 hospitalizations for HF. Between patients with and without the primary outcome, significant differences were observed in terms of mean arterial pressure (MAP), serum sodium (sNa), log transformed NT-proBNP [ln(NT-proBNP)], and presence of LGE. Regarding LV myocardial strain, none of the systolic strain parameters differed significantly between groups.

### Survival analysis

**Fig. 3:**
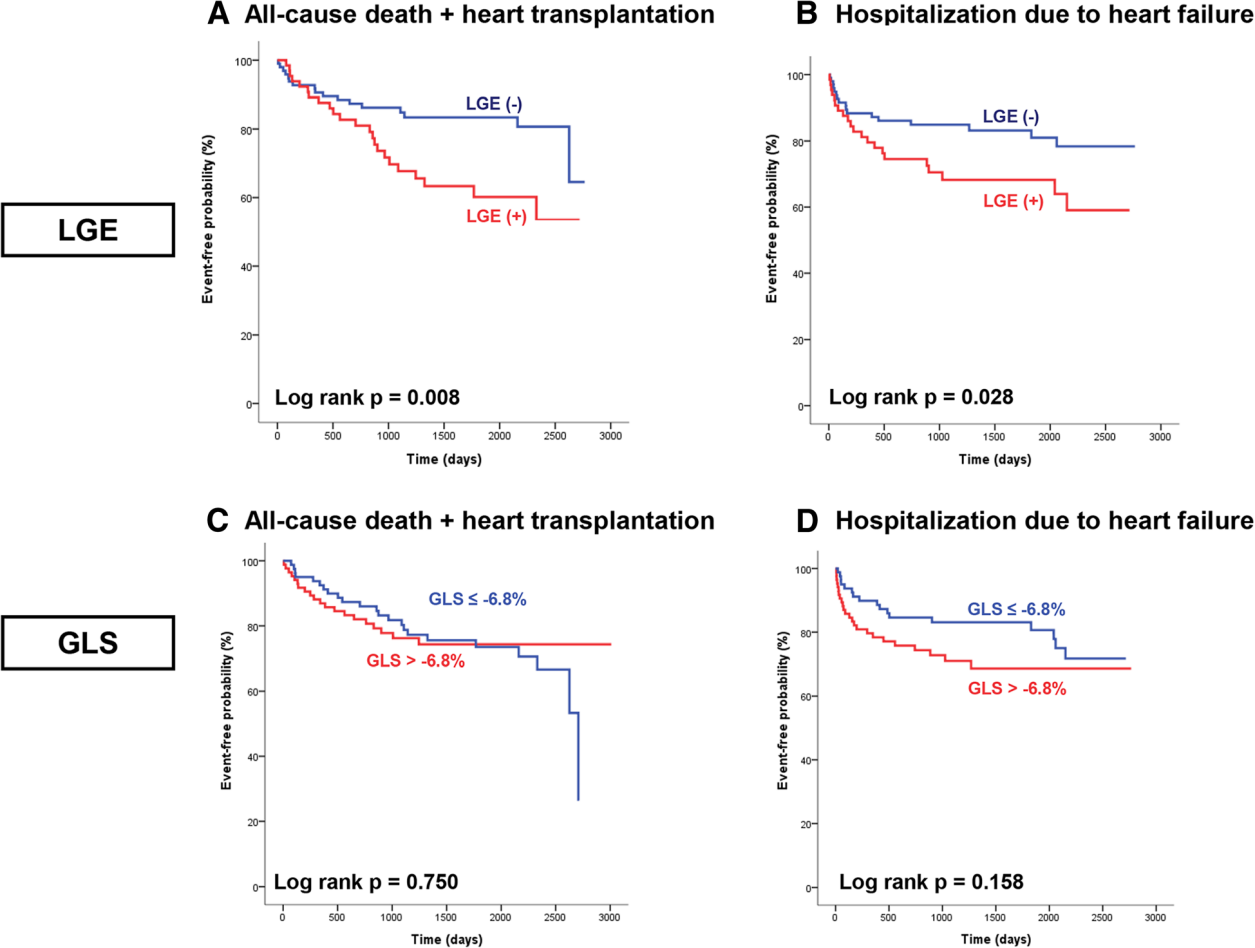
Kaplan-Meier Analysis of Clinical Outcomes According to Presence of Late Gadolinium Enhancement (LGE) and Global Longitudinal Strain (GLS). Kaplan-Meier curves are shown for (**a**, **c**) primary outcome and (**b**, **d**) secondary outcome according to presence of LGE and GLS. Patients were divided into two groups according to the median GLS of the total population (− 6.8%). Abbreviations: GLS, global longitudinal strain; LGE, late gadolinium enhancement

By univariate analysis, the following clinical parameters were predictors of the primary outcome: presence of LGE, MAP, history of cerebrovascular accident, Na, ln(NT-proBNP), RV end-diastolic volume (EDV), and RV end-systolic volume (ESV). The presence of LGE, MAP, sNa, ln(NT-proBNP), LV ESV, RV EF, RV ESV, and LV mass were all predictors of the secondary outcome. LV myocardial strain had no significant association with either the primary or secondary outcome (Table 3). Kaplan-Meier curves for the clinical outcomes according to presence of LGE and global longitudinal strain (GLS) are shown in Fig. 3. In Fig. 3c, d, patients were divided into two groups by the median GLS of the total population (− 6.8%).

**Table 3:** Univariate analysis of primary and secondary outcomes

	*Primary outcome*			*Secondary outcome*		
	*HR*	*95% CI*	*P value*	*HR*	*95% CI*	*P value*
Age (years)	0.993	0.973–1.013	0.487	0.991	0.970–1.011	0.373
Male gender	1.350	0.693–2.631	0.378	0.726	0.388–1.361	0.318
Mean arterial pressure (mmHg)	0.971	0.945–0.997	0.028	0.959	0.931–0.986	0.004
Hypertension	1.257	0.663–2.384	0.483	0.896	0.449–1.789	0.756
Diabetes mellitus	1.417	0.712–2.818	0.321	1.387	0.679–2.833	0.369
Dyslipidemia	1.521	0.469–4.936	0.485	0.945	0.228–3.917	0.938
Current smoker	0.978	0.474–2.017	0.951	0.853	0.403–1.807	0.678
Chronic kidney disease^a^	1.660	0.813–3.389	0.164	1.709	0.837–3.492	0.141
Previous CVA	3.501	1.079–11.362	0.037	2.265	0.545–9.418	0.261
Body mass index, kg/m^2^	0.960	0.888–1.037	0.298	0.936	0.858–1.021	0.134
ECG at baseline						
Heart rate (bpm)	1.000	0.984–1.015	0.972	1.013	0.997–1.029	0.109
Left bundle-branch block	1.122	0.519–2.426	0.770	1.580	0.774–3.224	0.209
QRS duration (ms)	1.005	0.995–1.015	0.353	1.003	0.993–1.013	0.598
Laboratory data						
Serum creatinine (mg/dl)	2.249	0.784–6.455	0.132	0.950	0.271–3.334	0.950
Na (mmol/l)	0.810	0.751–0.875	< 0.001	0.860	0.794–0.931	< 0.001
ln(NT-proBNP) (pg/ml)	1.376	1.083–1.748	0.009	1.519	1.190–1.939	0.001
Cardiac medications						
Beta-blockers	1.148	0.564–2.334	0.704	0.767	0.396–1.486	0.432
ACE-inhibitors/ARB	1.492	0.531–4.192	0.447	2.171	0.669–7.050	0.197
Spironolactone	1.100	0.588–2.056	0.766	1.380	0.724–2.632	0.328
Diuretics	0.921	0.463–1.832	0.814	1.732	0.767–3.914	0.187
Digoxin	1.658	0.847–3.242	0.140	1.662	0.847–3.258	0.140
LV EF (%)	1.006	0.969–1.044	0.759	0.970	0.933–1.009	0.132
LV EDV (ml)	1.003	1.000–1.006	0.093	1.003	1.000–1.006	0.090
LV ESV (ml)	1.003	0.999–1.006	0.108	1.004	1.000–1.007	0.041
Cardiac output (L/min)	1.092	0.908–1.314	0.349	0.961	0.784–1.177	0.699
Cardiac index (L/min/m^2^)	1.228	0.868–1.738	0.245	1.024	0.708–1.481	0.901
RV EF (%)	0.984	0.964–1.005	0.124	0.976	0.955–0.998	0.033
RV EDV (ml)	1.005	1.000–1.009	0.041	1.004	0.999–1.009	0.080
RV ESV (ml)	1.005	1.000–1.009	0.049	1.005	1.000–1.010	0.045
RV stroke volume (ml)	1.007	0.991–1.024	0.376	0.995	0.977–1.012	0.556
RV cardiac output (L/min)	1.122	0.894–1.407	0.320	0.962	0.759–1.220	0.751
Presence of LGE	2.277	1.221–4.246	0.010	2.023	1.066–3.839	0.031
Myocardial mass (g)	0.997	0.990–1.005	0.510	0.987	0.978–0.997	0.008
Quantitative LGE mass (g)	1.014	0.998–1.031	0.086	1.009	0.990–1.028	0.370
LGE mass/LV myocardial mass (%)	1.024	0.998–1.051	0.068	1.018	0.989–1.047	0.232
Global radial strain (%)	0.974	0.921–1.030	0.363	0.955	0.901–1.013	0.129
Global circumferential strain (%)	1.030	0.934–1.136	0.554	1.062	0.963–1.172	0.229
Global longitudinal strain (%)	1.048	0.945–1.163	0.375	1.066	0.955–1.191	0.254

^a^Chronic kidney disease was defined as eGFR < 60 ml/min/1.73m^2^, calculated using the 4-component MDRD study equation

Abbreviations: *ACE* angiotensin-converting-enzyme, *ARB* angiotensin II receptor blockers, *BNP* B-type natriuretic peptide, *CI* confidence interval, *CVA* cerebrovascular accident, *ECG*, electrocardiography, *EDV* end-diastolic volume, EF, ejection fraction, *ESV*, end-systolic volume, *HR*, hazard ratio, *LGE* late gadolinium enhancement, *LV*, left ventricle, *RV*, right ventricle

Multivariable analysis showed that presence of LGE and sNa were independently associated with the primary outcome and presence of LGE, sNa, myocardial mass and ln(NT-proBNP) were independent predictors for secondary outcome. However, the effect of LGE did not satisfy the proportional hazard assumption for the primary outcome, and as stated in the Statistical methods section, we accommodate the LGE effects as time-dependent. The best model indicated that the effect of LGE was significant and dramatically apparent after about 6 months (Table 4).

**Table 4:** Multivariable proportional-hazard model of primary and secondary outcomes

	*Primary outcome* ^a^			*Secondary outcome*		
	*HR*	*95% CI*	*P value*	*HR*	*95% CI*	*P value*
Na (mmol/l)	0.821	0.761–0.885	< 0.001	0.896	0.817–0.982	0.019
Presence of LGE	4.729	1.111–20.121	0.0355	2.358	1.229–4.523	0.010
Myocardial mass				0.986	0.975–0.996	0.009
lnNT-proBNP				1.352	1.031–1.771	0.029

^a^Based on a multivariable Cox model with time-dependent covariates at 6 months. Before 6 months, the HR was 0.71 (*p*-value = 0.58, 95% CI: 0.206, 2.415), while after 6 months, the HR is as given in the Table

The Harrell’s c-index of multivariable Cox proportional hazards model were 0.727 (95% CI: 0.616 to 0.838) and 0.744 (95% CI: 0.648 to 0.839) for primary and secondary outcomes, respectively

Abbreviations: NT-proBNP, N-terminal pro-B-type natriuretic peptide; *CI* confidence interval, *HR* hazard ratio, *LGE* late gadolinium enhancement

### Reliability

Intra- and inter-observer reliability values were excellent. Intra- and inter-observer intraclass correlation coefficients were 0.993 (95% CI: 0.985–0.997) and 0.944 (95% CI: 0.875–0.975), respectively, for GLS; 0.993 (95% CI: 0.985–0.997) and 0.962 (95% CI: 0.915–0.983), respectively, for global circumferential strain (GCS); and 0.986 (95% CI: 0.971–0.993) and 0.955 (95% CI: 0.899–0.980), respectively, for global radial strain (GRS).

## Discussion

In this study, we evaluated the clinical outcomes of idiopathic DCM patients with moderate to severe LV systolic dysfunction according to CMR-derived LV strain and LGE. Our major finding was that LV strain had no significant prognostic value in these high-risk patients while the presence of LGE was a significant predictor of adverse outcomes.

### Differential prognosis according to LV strain

To the best of our knowledge, a study published by Buss et al. has been the only other study that evaluated the prognostic value of CMR-derived LV strain in the DCM population [24]. In contrast to our study, they found that CMR-derived LV longitudinal strain was an independent predictor of survival in DCM that offered incremental information for risk stratification beyond clinical parameters, biomarkers, and standard CMR. However, they included patients with various systolic functions, and the LV EF of their study population was 36.1 ± 13.8%, much higher than that in our study (23.7 ± 7.9%).

LV myocardial strain is inevitably related to LV EF, and it has been reported that LV EF is determined by global myocardial strain and myocardial thickness [29]. In our study, the Pearson correlation coefficients between LV EF and GLS, GCS, and GRS were − 0.733 (*P* < 0.01), − 0.780 (*P* < 0.01), and 0.739 (*P* < 0.01), respectively. LV EF was not associated with the outcomes in our study, as expected because all of our study patients had severely depressed LV EF. Likewise, it is not surprising that LV strain could not predict adverse outcomes because it is so closely related to LV EF.

### Differential prognosis according to LGE

Regarding the differential clinical outcomes according to LGE, previous studies have well demonstrated this correlation in patients with DCM. It has been reported that presence of LGE was associated with adverse clinical outcomes such as cardiovascular death, hospitalization due to HF, and sudden death [20–23, 30]. This study showed results similar to those of previous studies: the presence of LGE was the strong independent predictor for adverse outcomes. We also considered the quantitative extent of LGE, but the extent of LGE was not significantly related to clinical outcomes. The presence of LGE might thus be more important than the extent of LGE when predicting adverse outcomes.

### Limitations

This study has some important limitations. First, there are inherent limitations in non-randomized comparisons, such as allocation bias, uneven distribution of risk factors, and the possibility of unmeasured confounders. Second, data regarding the cause of death such as cardiovascular death including pump failure and sudden cardiac death was not available. Third, the follow-up duration varied by individual patient. Next limitation was that those with several forms of reversible cardiomyopathies such as tachycardia induced cardiomyopathy or hypertensive heart failure were excluded. Furthermore, many patients were excluded from the analysis of LV strain. Since this might have resulted in selection bias, so the findings of our study would not be generalizable to the entire HF with reduced EF population. However, there was no difference in the presence of LGE which was one of main CMR variable between those who were included in the strain analysis and excluded patients. Thus the overall finding might be not affected. Lastly, HF management was not controlled; thus, our conclusions should not be extrapolated to all patients with idiopathic DCM. A further study with a prospective and multicenter design is required.

## Conclusions

In idiopathic DCM patients with reduced EF, CMR LGE is a good predictor of adverse outcomes. LV strain, however, had no significant prognostic value in patients with moderate to severe systolic dysfunction. As our study was analyzed retrospectively and selection bias could not be excluded, further studies with prospective design are warranted to support our findings.

## Additional file

Additional file 1: **Table S1.** Baseline characteristics between 172 included and 95 excluded patients. **Table S2.** Baseline standard CMR-data between 172 included and 95 excluded patients. (PDF 149 kb)

## Abbreviations

ACE: Angiotensin converting enzyme inhibitor
ARB: Angiotensin receptor blocker
CMR: Cardiovascular magnetic resonance
DCM: Dilated cardiomyopathy
EDV: End diastolic volume
EF: Ejection fraction
ESV: End-systolic volume
GCS: Global circumferential strain
GLS: Global longitudinal strain
GRS: Global radial strain
HF: Heart failure
HR: Hazard ratio
LGE: Late gadolinium enhancement
LV: Left ventricle/Left ventricular
MAP: Mean arterial pressure
RV: Right ventricle/Right ventricular
SD: Standard deviation
sNA: Serum sodium
