# Supplementary material for: Prognostic value of myocardial strain and late gadolinium enhancement on cardiovascular magnetic resonance imaging in patients with idiopathic dilated cardiomyopathy with moderate to severely reduced ejection fraction
Source: J Cardiovasc Magn Reson. 2018 Jun 14;20:36. doi: 10.1186/s12968-018-0466-7 (PMC6001169; doi:10.1186/s12968-018-0466-7)
Supplement: Supplementary file 1 — Table S1. Baseline characteristics between 172 included and 95 excluded patients. Table S2. Baseline standard CMR-data between 172 included and 95 excluded patients. (PDF 149 kb) [file 12968_2018_466_MOESM1_ESM.pdf]

Additional file 1

This appendix has been provided by the authors to give readers additional information about their work.

Prognostic value of myocardial strain and late gadolinium enhancement on cardiac magnetic resonance imaging in patients with idiopathic dilated cardiomyopathy with severely reduced ejection fraction

Seung-Hoon Pi, MD[1,*], Sung Mok Kim, MD, PhD[2,*], Jin-Oh Choi, MD, PhD[1], Eun Kyoung Kim, MD, PhD[1], Sung-A Chang, MD, PhD[1], Yeon Hyeon Choe, MD, PhD[2], Sang-Chol Lee, MD, PhD[1], Eun-Seok Jeon, MD, PhD[1]

Table of content of the Additional file 1

I. Supplementary Tables

I. Supplementary Tables

Table S1 Baseline characteristics between 172 included and 95 excluded patients.

Parameters	*Patients who included (n=172)*	*Patients who excluded (n=95)*	*P value*
Age (years)	56.4 ± 14.3	57.7 ± 14.0	0.477
Male gender, n (%)	116 (67.4)	70 (73.7)	<0.001
Mean arterial pressure (mmHg)	84 ± 13	85 ± 13	0.734
Hypertension, n (%)	58 (33.7)	31 (32.6)	0.857
Diabetes mellitus, n (%)	36 (20.9)	15 (15.8)	0.306
Dyslipidemia, n (%)	11 (6.4)	3 (3.2)	0.256
Current smoker, n (%)	49 (28.5)	31 (32.6)	0.738
Chronic kidney disease*, n (%)	31 (18.0)	16 (16.8)	0.808
Previous CVA, n (%)	4 (2.3)	6 (6.3)	0.100
Body mass index (kg/m^2)	24.0 ± 4.5	23.4 ± 3.9	0.220
ECG at baseline			
Heart rate (bpm)	83 ± 20	79 ± 18	0.061
Left bundle-branch block, n (%)	31 (18.0)	5 (5.3)	0.003
QRS duration (ms)	113 ± 29	108 ± 25	0.144
Laboratory data			
Serum creatinine (mg/dl)	0.97 ± 0.27	1.01 ± 0.31	0.247

Na (mmol/l)	139.6 ± 3.3	139.0 ± 3.3	0.218
ln(NT-proBNP) (pg/ml)	7.22 ± 1.27	7.25 ± 1.20	0.809
Cardiac medications			
Beta-blockers, n (%)	120 (69.8)	57 (60.0)	0.106
ACE-inhibitors/ARB, n(%)	143 (83.1)	82 (86.3)	0.495
Spironolactone, n (%)	101 (58.7)	58 (61.1)	0.710
Diuretics, n (%)	125 (72.7)	75 (78.9)	0.258
Digoxin, n (%)	33 (19.2)	31 (32.6)	0.014

Primary outcome: all-cause death, heart transplantation during follow-up. Values are mean ± SD, n(%).

*Chronic kidney disease was defined as eGFR < 60ml/min/1.73m2, calculated using the 4-component MDRD study equation.

Abbreviations: ACE, angiotensin-converting-enzyme; ARB, angiotensin II receptor blockers; BNP, B-type natriuretic peptide; CVA, cerebrovascular accident; ECG, electrocardiography

Table S2 Baseline standard CMR-data between 172 included and 95 excluded patients.

Parameters	*Patients who included (n=172)*	*Patients who excluded (n=95)*	*P value*
LV EF (%)	23.7 ± 7.9	24.8 ± 7.3	0.269
LV EDV (ml)	284.3 ± 91.4	264.6 ± 96.3	0.099
LV ESV (ml)	219.9 ± 85.7	201.2 ± 83.2	0.087
Cardiac output (L/min)	5.04 ± 1.53	5.05 ± 2.44	0.960
Cardiac index (L/min/m^2)	2.95 ± 0.84	2.96 ± 1.31	0.916
RV EF (%)	41.2 ± 17.0	39.2 ± 13.4	0.342
RV EDV (ml)	145.6 ± 60.7	156.4 ± 66.6	0.184
RV ESV (ml)	91.6 ± 56.7	100.4 ± 59.1	0.232
RV cardiac output (L/min)	4.21 ± 1.33	4.36 ± 1.50	0.422
Presence of LGE, n (%)	66 (38.4)	43 (47.3)	0.215
Myocardial mass (g)	142.2 ± 41.1	129.5 ± 37.3	0.014
Quantitative LGE mass (g)	6.8 ± 14.5	5.7 ± 10.2	0.521
LGE mass/LV myocardial mass (%)	4.7 ± 9.5	4.5 ± 7.6	0.845

Primary outcome: all-cause death, heart transplantation. Values are mean ± SD, n(%).

Abbreviations: CMR, cardiac magnetic resonance; EDV, end-diastolic volume; EF, ejection fraction; ESV, end-systolic volume; LGE, late gadolinium enhancement; LV, left ventricle; RV, right ventricle
